# 

**Published:** 1883-12

**Authors:** 


ORIGINAL COMMUNICATIONS:

Caries of the Human Teeth. W. D. Miller.......................... 629
American Dental Association. K.—H.......................... 645
Gutta-Percha Stopping. C. E. Francis.................. 649
A Case in Practice. W. P. Richards.......................... 650
Notes on Operative Dentistry and its Advancement. Geo. H. Perine... 652

REPORTS OF SOCIETY MEETINGS:

American Dental Society of Europe.......................... 656
Ohio State Dental Society.......................... 661
Connecticut Valley Dental Society.......................... 667

CORRESPONDENCE.......................... 671

EDITORIAL:

Dental Education.......................... 675
Retrospective and Prospective.......................... 677
Dr. Miller's Latest Investigation.......................... 679
Is Dental Caries a Modern Disease.......................... 680
To Our Subscribers.......................... 681
Credit to Whom Credit is Due.......................... 682
Visiting Lists.......................... 682
Hear Both Sides.......................... 682
Adding Strength to Strength.......................... 683
Respect Our Identity.......................... 683
Combined Filling.......................... 683

CURRENT NEWS AND OPINION:

Dr. Gregg vs. Bacteria.......................... 683
Oral Pathology.......................... 685
American Academy of Dental Science.......................... 685
Damages for Accidents During Anæsthesia.......................... 686
Dr. Lord's Instruments.......................... 686
An Irresponsible Call.......................... 686
A Righteous Judgment.......................... 687
Dio Lewis' Monthly.......................... 687
Lyon's Tooth Powder.......................... 687
To Remove Warts.......................... 687
Good Solders.......................... 648
Right-handedness.......................... 644

